# Application of Box–Behnken Design to Investigate the Effect of Process Parameters on the Microparticle Production of Ethenzamide through the Rapid Expansion of the Supercritical Solutions Process

**DOI:** 10.3390/pharmaceutics12010042

**Published:** 2020-01-03

**Authors:** Yung-Tai Hsu, Chie-Shaan Su

**Affiliations:** Department of Chemical Engineering and Biotechnology, National Taipei University of Technology, Taipei 10608, Taiwan

**Keywords:** supercritical, RESS, microparticle, ethenzamide, Box–Behnken design

## Abstract

In this study, the rapid expansion of the supercritical solutions (RESS) process was used to produce microparticles of a commonly used anti-inflammatory drug, ethenzamide. The effects of process parameters in RESS including the extraction temperature, pre-expansion temperature, and post-expansion temperature were investigated using the Box–Behnken design. According to the results of the analysis of variance (ANOVA), the effect of pre-expansion temperature is the most significant parameter on the mean size of RESS-produced ethenzamide. A higher pre-expansion temperature benefits the production of smaller crystals. In addition, a quadratic effect of the post-expansion temperature was also identified. Through RESS, ethenzamide microparticles with a mean size of 1.6 μm were successfully produced. The solid-state properties including the crystal habit, crystal form, thermal behavior, and spectrometric property were characterized by scanning electron microscopy (SEM), Fourier-transform infrared spectrometer (FTIR), differential scanning calorimeter (DSC), and powder X-ray diffraction (PXRD). These analytical results show that the rod-like crystals were generated through RESS, and the crystal form, thermal behavior, and spectrometric property of RESS-produced crystals are consistent with the unprocessed ethenzamide.

## 1. Introduction

Microparticle production is a strategy used in the pharmaceutical industry to enhance the dissolution profile and improve the bioavailability of poorly water-soluble substances, especially for the biopharmaceutics classification system’s (BCS) class II and IV active pharmaceutical ingredients [1,2,3]. Supercritical fluid technology is one of the intensified particle design methods to produce microparticles of pharmaceutical solids to overcome disadvantages such as thermal degradation, broad particle size distribution, and residual solvent contamination in conventional route [4,5,6,7]. According to the role of the supercritical fluid used, different processes such as the rapid expansion of supercritical solutions (RESS), supercritical antisolvent (SAS), particles from gas-saturated solutions (PGSS), and supercritical-assisted atomization (SAA) were proposed in the literature [8,9]. Among these techniques, the rapid expansion of supercritical solutions (RESS) is an organic solvent-free process, beneficial in the particle formation of pharmaceutical solids. In RESS, the supercritical fluid is used as the solvent to extract the pharmaceutical solid packed in an extraction vessel at a high pressure. The supercritical solution is subsequently depressurized to an atmospheric condition through a nozzle, to achieve a high supersaturation for particle generation. In the literature, RESS has been successfully adopted for producing microparticles of pharmaceutical compounds such as olanzapine, mefenamic acid, nabumetone, paracetamol, tolbutamide, lonidamine, vanillin, and coumarin [10,11,12,13,14]. 

In this study, the RESS process was adopted for microparticle production of ethenzamide. Ethenzamide is a commonly used non-steroidal anti-inflammatory drug (NSAID), which is used as an analgesic and antipyretic agent for pain relief [15,16]. According to the PubChem database, ethenzamide is a poorly water-soluble drug, and its water solubility is less than 1 mg/mL at 289 K. The poor aqueous solubility of ethenzamide results in poor dissolution, poor absorption, and low bioavailability, and makes drug formulation more difficult. Thus, particle design and crystal engineering of ethenzamide are necessary to develop a pharmaceutical formulation and improve the drug’s efficacy. For example, Danjo et al. prepared the solid dispersion of ethenzamide using various sugars as dispersion carriers to improve the dissolution behavior [17]. Kozak et al. designed three novel ethenzamide cocrystals and then characterized their pharmaceutical properties [18]. In addition, ethenzamide is also a commonly used model compound in the literature, to demonstrate the feasibility of novel processes for dissolution improvement. For example, Fukunaka et al. produced micronized ethenzamide using a fluidized-bed jet milling [19]. Ozawa et al. prepared the solid dispersion of ethenzamide by the twin screw extruder method and investigated the dissolution profile [20]. Mizoe et al. studied the use of a 4-fluid nozzle spray drier for producing drug-containing microparticles to improve the dissolution of ethenzamide [21].

In this RESS study, the Box–Behnken design was used to systematically study the effect of the process parameter on the mean size of ethenzamide crystals. The Box–Behnken design is a response surface methodology and can be used for representing a response function that cannot be described by linear functions. Compared with other response surface methodologies, such as the three-level factorial design, the Box–Behnken design is more economical and efficient for a large number of variables, and has been widely used in analytical chemistry and energy applications [22,23]. To screen the significant factors and the appropriate operating range in the Box–Behnken design, a preliminary RESS study was conducted firstly by the one-factor-at-a-time approach. Furthermore, the crystal habit, crystal form, thermal behavior, and spectrometric property of unprocessed and RESS-produced ethenzamide were analyzed and compared through scanning electron microscopy (SEM), powder X-ray diffraction (PXRD), differential scanning calorimetry (DSC), and Fourier-transform infrared spectrometry (FTIR).

## 2. Materials and Methods

**Figure 1:**
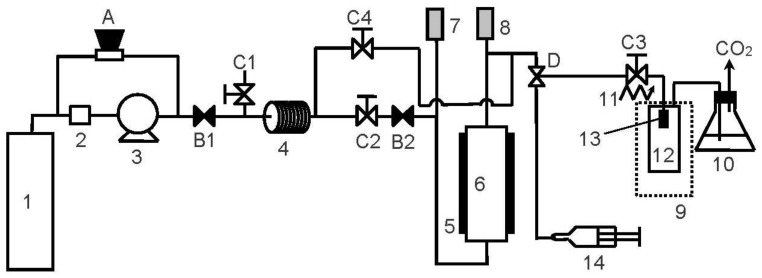
Experimental apparatus of the rapid expansion of the supercritical solutions (RESS) process (1: CO_2_ cylinder; 2: cooler; 3: CO_2_ pump; 4: preheating line; 5: electric heater; 6: extraction vessel; 7: pressure transducer; 8: thermocouple; 9: water bath; 10: cold trap; 11: heating tape; 12: expansion vessel; 13: nozzle; 14: syringe; A: back-pressure regulator; B: check valves; C: two-way needle valves; D: three-way needle valve).

In the RESS operation, supercritical carbon dioxide (CO_2_) was purchased from Cheng Feng Gas Co., Taipei, Taiwan with a purity of 99.5%. The active pharmaceutical ingredient, ethenzamide (CAS: 938-73-8), was supplied by Tokyo Chemical Industry Co., Tokyo, Japan with a purity of 99.6%. All chemicals were used without further purification. The schematic diagram of the RESS process is shown in Figure 1. This apparatus has been used for microparticle production of diuron in our previous work [24]. The RESS apparatus comprises three sections for CO_2_ supply, solute extraction, and particle generation. In the CO_2_ supply section, CO_2_ from the gas cylinder (1) was liquefied and pressurized by a cooler (2) and a high-pressure pump (3). The pressure of the CO_2_ stream was manipulated by a back-pressure regulator (A). The pressurized CO_2_ stream was then entered into the solute extraction section for dissolving ethenzamide through a preheating line (4) and extraction cell (6). The temperature of the extraction cell was regulated by an electric heater (5). The extraction pressure and temperature were recorded by a pressure transducer (7) and a thermometer (8) with resolutions of 0.1 MPa and 0.1 K, respectively. The high-pressure CO_2_ stream containing dissolved ethenzamide was then expanded into an atmospheric pressure through a 50 μm capillary nozzle (13) in the particle generation section. Prior to expansion, the CO_2_ stream was heated by a heating tape (11) to attain a pre-expansion temperature to prevent the nozzle from clogging. To collect the generated ethenzamide microparticles, a glass vial was used as the expansion vessel (12) and its temperature was maintained by a heating/cooling circulator (9) at a post-expansion temperature. The produced ethenzamide microparticles were collected in the expansion vessel for further solid-state property characterization.

In this study, the solid-state properties, including the crystal habit, particle size, crystal form, thermal behavior, and spectrometric property of ethenzamide crystals, were compared and discussed. The crystal habit of ethenzamide was characterized by a scanning electron microscope (Hitachi S-3000H, Taipei, Taiwan). The ethenzamide samples were attached to a sample holder using a double-side tape and sputter-coated with gold by a sputter coater at room temperature, prior to the SEM analysis. The particle size characteristics of ethenzamide crystals were further calculated by counting the length of ethenzamide crystals from SEM images using the software Image J, NIH, Maryland, USA. The mean particle size and size distribution were calculated for at least 150 particles. The same procedure to acquire particle size characteristics from SEM images was also adopted in the literature for the particle design of raloxifene and diclofenac sodium using supercritical fluid technology [25,26]. A Fourier-transform infrared spectrometer (FTIR) instrument (PerkinElmer, Taipei, Taiwan, Spectrum 100), differential scanning calorimeter (DSC) instrument (PerkinElmer, Taipei, Taiwan, DSC 4000), and powder X-ray diffraction (PXRD) instrument (PANalytical X’pert^3^ Powder) were used to analyze the spectrometric property, thermal behavior, and crystal form of ethenzamide samples, respectively. For DSC measurements, a 1–2 mg sample was heated with a single heating range of 50–135 °C at 10 °C/min in an aluminum-made standard pan to determine the melting point (onset temperature) and heat of fusion. A sealed empty pan was used as reference. The nitrogen purge flow rate of 20 mL/min was used, and the DSC instrument was calibrated using indium and zinc. The FTIR spectra of ethenzamide were collected within a wavenumber of 450–4000 cm^−1^ with resolution of 4 cm^−1^, using an attenuated total reflection sample holder. Transmission mode spectra were taken at room temperature. The PXRD pattern was collected from 10°–50° at a scanning rate of 52°/min.

## 3. Results and Discussion

To produce microparticles of ethenzamide and systematically investigate the significance of the process parameter in RESS, the Box–Behnken design was used. The Box–Behnken design is a response surface method that is suitable for investigating quadratic response surfaces and developing second-order polynomial models. It has been used to investigate the effect of the process parameter in supercritical processes such as supercritical fluid extraction [27,28] and supercritical fluid particle design [25,29,30]. According to the RESS studies reported in the literature [10,11,12,13,14], the solid-state property of generated microparticles can be efficiently manipulated by process parameters such as the extraction temperature, extraction pressure, pre-expansion temperature, post-expansion temperature, nozzle design, and spraying distance. In this RESS study, a 50 μm capillary nozzle with a spraying distance of 5 cm was used and fixed, owing to the limitation of the experimental system. To further screen the process parameter and select its range in the Box–Behnken design, a preliminary one-factor-at-a-time RESS study was conducted for investigating the effect of extraction temperature, extraction pressure, pre-expansion temperature, and post-expansion temperature. The operation conditions and results of this preliminary RESS study are presented in the Supporting Information as Table S1 and Figure S1. The ranges of extraction temperature, extraction pressure, pre-expansion temperature, and post-expansion temperature are designed with reference to the literature [10,11,12,13,14]. From Table S1 and Figure S1, it was found that the effect of extraction pressure on the mean size of RESS-produced ethenzamide was negligible. Therefore, the process parameters including the extraction temperature, pre-expansion temperature, and post-expansion temperature were finally considered in the Box–Behnken design. The ranges of process parameters used in the Box–Behnken design were summarized in Table 1. The conditions of a total of 15 experiments in the Box–Behnken design with three replicates of the center point are listed in Table 2. The experimental results of the mean size and size distribution of produced crystals are also listed in Table 2. The particle size distribution of ethenzamide crystals was described by the standard deviation of size of counting particles. To select the approximate model to fit the mean size data, Table 3 shows the *R*^2^ value in data fitting and the *p*-value (Prob > F) in lack of fit test for the three considered models, including the linear, two-factor interactive (2FI), and quadratic models. As presented in Table 3, the quadratic model shows the maximum *R*^2^ in data fitting and the maximum *p*-value in lack of fit test. The lack of fit test indicates the failure of the model. The maximum *p*-value of the quadratic model revealed that this model is the most statistically significant. To further verify the significance of the quadratic model, Table 4 reports the analysis of variance (ANOVA) results of the model. As can be seen, the *p*-value of the quadratic model was 0.0025. The *p*-value lower than 0.05 indicates that the quadratic model is statistically significant and adequate to show the relationship between the responses and the variables. By adopting the quadratic model, an empirical equation was reported to calculate the mean size of RESS-produced crystals, as seen below.

Mean size (μm) = 9.22 + 1.66 × 10^−1^ A − 8.21 × 10^−2^ B + 3.30 × 10^−2^ C + 1.00 × 10^−3^ AB − 3.88 × 10^−4^ AC + 1.28 × 10^−4^ BC − 4.08 × 10^−3^ A^2^ + 1.67 × 10^−5^ B^2^ − 1.33 × 10^−3^ C^2^. (1)

The parameters A, B, and C are the actual value of the extraction temperature in °C, the pre-expansion temperature in °C, and the post-expansion temperature in °C, respectively. The comparison of experimental and calculated mean sizes are also listed in Table 2. The relative deviation of the model calculation was less than 15%.

**Figure 2:**
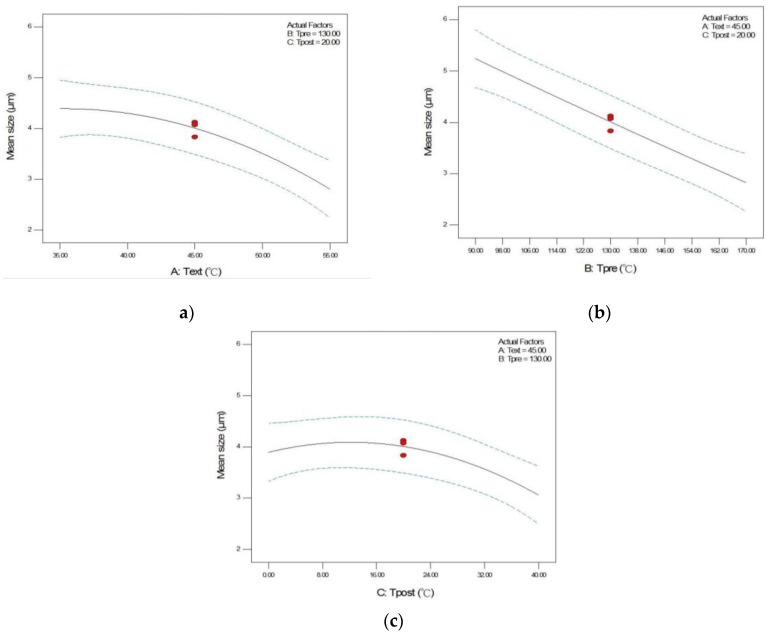
Effects of (**a**) extraction temperature (**b**) pre-expansion temperature, and (**c**) post-expansion temperature on the mean size of RESS-processed ethenzamide crystals using Box–Behnken design.

In addition to reporting the empirical model, the significance of the process parameters in RESS was also compared and discussed according to the ANOVA results listed in Table 4. It was found that the first-order effect of extraction temperature (A), pre-expansion temperature (B), and post-expansion temperature (C), and the second-order effect of post-expansion temperature (C^2^) are significant model terms. The other terms, including the two-level interaction of extraction temperature and pre-expansion temperature (AB), extraction temperature and post-expansion temperature (AC), and pre-expansion temperature and post-expansion temperature (BC), and the second-order effects of extraction temperature (A^2^) and pre-expansion temperature (B^2^) were insignificant but required in the empirical equation to provide model accuracy. The trend of the significant model terms on the mean size of RESS-processed crystals is shown in Figure 2. As shown in Figure 2a,b, a monotonically decrease in the mean size of ethenzamide crystals with the increase in the extraction temperature and pre-expansion temperature was observed. On the other hand, according to Figure 2c and the ANOVA results, the effect of post-expansion temperature (C) showed a quadratic trend (C^2^) on the mean size of RESS-processed crystals. Regarding the effect of extraction temperature, a higher extraction temperature is preferable for producing smaller ethenzamide crystals. Regarding the effect of extraction temperature, according to the solubility of a solid solute in supercritical fluid, at a higher extraction pressure the solubility value of the solid solute increased with the extraction temperature. The higher solubility resulted in a higher supersaturation, which then favored the production of smaller ethenzamide crystals. Regarding the effect of pre-expansion temperature, it was found that a higher pre-expansion temperature benefited the production of smaller crystals. During the expansion of the supercritical solution through a capillary nozzle, the temperature decreased considerably, owing to the Joule–Thomson effect. The temperature drop possibly contributed to an early nucleation of ethenzamide inside the capillary nozzle. The generated nuclei in the capillary nozzle may grow further and aggregate to a large particle in the expansion vessel. Therefore, a higher pre-expansion temperature to compensate the Joule–Thomson effect may prevent the early crystal nucleation inside the capillary nozzle and subsequently decrease the extent of crystal growth and agglomeration. The quadratic trend for the effect of post-expansion temperature can be explained by the kinetics of nucleation and crystal growth during particle formation. At a low post-expansion temperature region, the crystal growth rate may increase dominantly with the temperature and then produce crystals with a large mean size. On the other hand, by further increasing the post-expansion temperature to a higher temperature region, the temperature increase may accelerate the nucleation rate considerably and benefit the generation of small crystals. 

**Figure 3:**
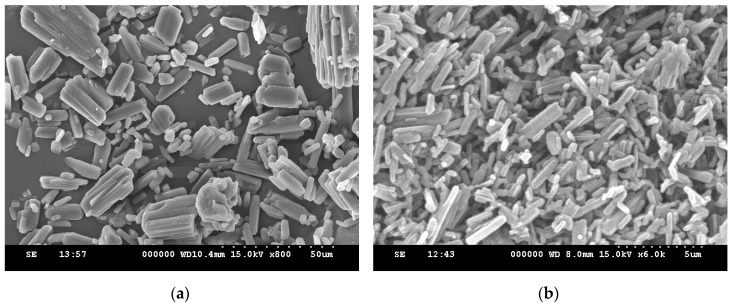
SEM images of (**a**) unprocessed ethenzamide and (**b**) RESS-processed ethenzamide from Experiment 12.

**Figure 4:**
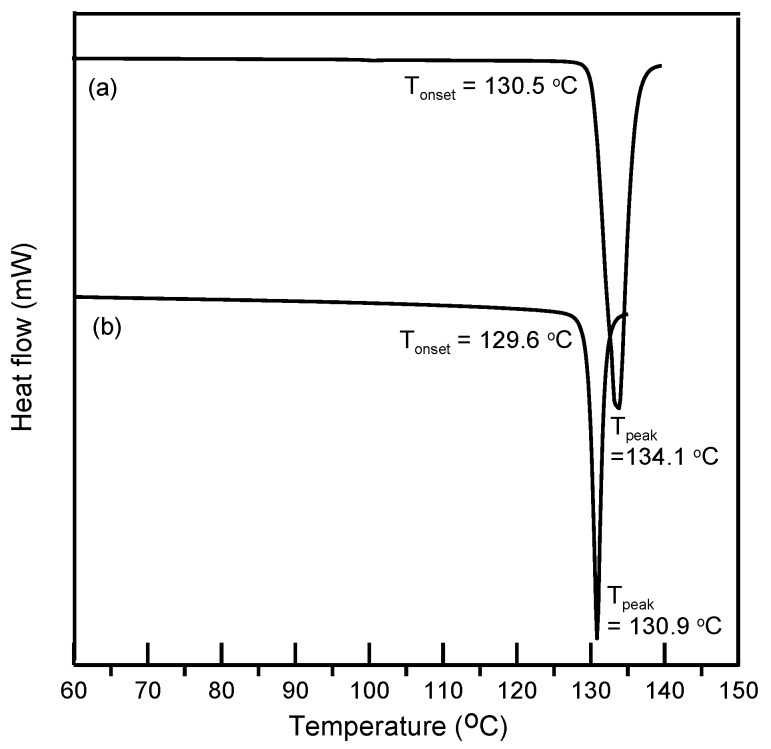
Differential scanning calorimeter (DSC) thermogram of (**a**) unprocessed ethenzamide and (**b**) RESS-processed ethenzamide from Experiment 12.

**Figure 5:**
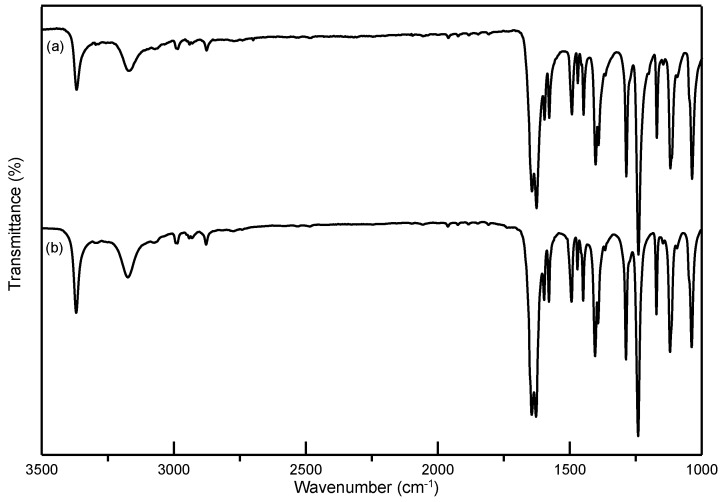
Comparison of FTIR spectra of (**a**) unprocessed ethenzamide and (**b**) RESS-processed ethenzamide from Experiment 12.

**Figure 6:**
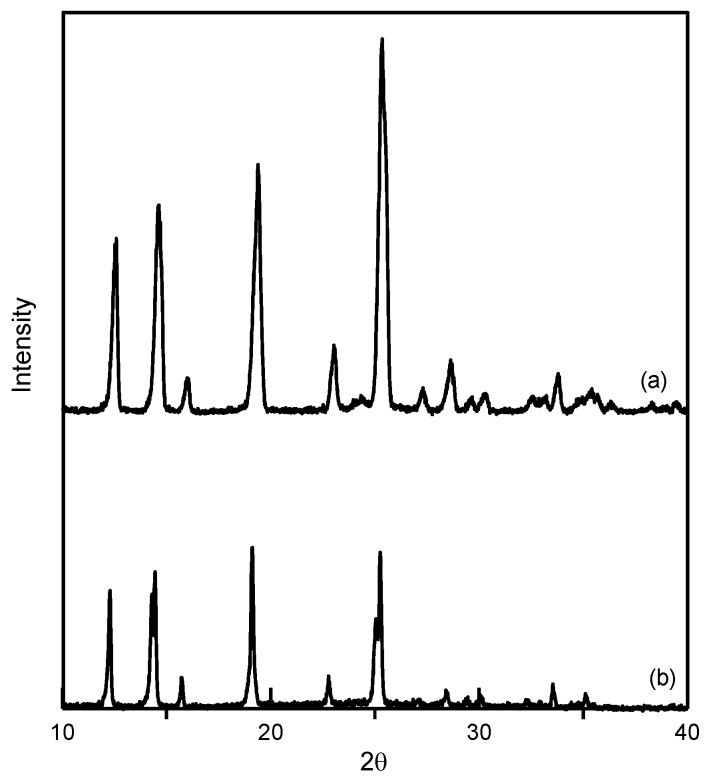
Comparison of powder X-ray diffraction (PXRD) patterns of (**a**) unprocessed ethenzamide and (**b**) RESS-processed ethenzamide from Experiment 12.

In this study, ethenzamide microparticles were successfully produced through RESS. In addition to the mean size, crystal habit and particle size distribution are also important to influence the powder quality and drug efficacy. Figure 3 compares the crystal habit of unprocessed ethenzamide and the RESS-processed sample from Experiment 12. As can be seen, the original ethenzamide exhibited an irregular shape and a relatively large mean size of 15.4 μm. After RESS, the mean size of ethenzamide had decreased to 1.6 μm and exhibited a rod-like habit. Figure S2 in the Supporting Information compares the particle size distributions of unprocessed ethenzamide and the RESS-processed ethenzamide from Experiment 12. Accordingly, the particle size distribution of ethenzamide crystals decreased considerably from the original 5–32 μm to the RESS-processed 1–3 μm. Furthermore, the crystal form, thermal behavior, and spectrometric property of the original and RESS-processed ethenzamide were investigated through PXRD, DSC, and FTIR. Figure 4 compares the DSC thermograms of ethenzamide crystals before and after RESS. The melting temperatures of the original and processed ethenzamide were consistent at about 130 °C. However, a sharper endothermic signal was observed in DSC thermograms for processed ethenzamide (T_peak_ = 130.9 °C), when compared with the unprocessed sample (T_peak_ = 134.1 °C). This observation may be attributable to the particle size reduction of ethenzamide that results in accelerated melting. In addition, according to the heat of fusion results obtained from DSC thermograms, the heat of fusion of the RESS-processed sample decreased about 10%–30% when compared with the unprocessed ethenzamide. The decrease in heat of fusion indicates a loss of crystallinity through RESS. Figure 5 presents a comparison of the FTIR spectra of the unprocessed ethenzamide and the RESS-produced particle from Experiment 12. As can be seen, the FTIR spectra of both samples are identical and indicate that the spectrometric properties of ethenzamide before and after RESS were consistent. Finally, Figure 6 compares the PXRD patterns of the unprocessed and RESS-processed ethenzamide. According to the Cambridge Crystallographic Data Centre (CCDC) database, ethenzamide has one crystal form (CCDC number: 760137), and the calculated powder pattern is shown in Figure S3 in the Supporting Information. According to Figure 6 and Figure S3, the PXRD pattern of RESS-processed ethenzamide (Experiment 12) is consistent with that of the original sample and identical to the evidence reported in the literature. This observation indicates that there was no polymorphic conversion during RESS processing.

## 4. Conclusions

This study investigates the effect of extraction temperature, pre-expansion temperature, and post-expansion temperature in the RESS process using the Box–Behnken design for microparticle production of an NSAID, ethenzamide. A quadratic model to describe the relationship between the mean size of ethenzamide crystals and the considered process parameter was reported. According to the ANOVA results, the first-order effect of extraction temperature, pre-expansion temperature, and post-expansion temperature, and the second-order effect of post-expansion temperature are significant. Operation at a higher extraction temperature and a higher pre-expansion temperature yielded smaller ethenzamide crystals, while the effect of post-expansion temperature showed a quadratic effect. In addition, this study produced rod-like ethenzamide crystals with a mean particle size as low as 1.6 μm. The RESS-produced ethenzamide crystals also showed consistent DSC, FTIR and PXRD results when compared with the unprocessed sample.

## Figures and Tables

**Table 1:** Factor levels of RESS Box–Behnken design.

Independent Variable	Symbol	Level		
		−1	0	1
A: Extraction temperature	T_ext_ (°C)	35	45	55
B: Pre-expansion temperature	T_pre_ (°C)	90	130	170
C: Post-expansion temperature	T_post_ (°C)	0	20	40

**Table 2:** Operating conditions and results of RESS Box–Behnken design ^(a)^.

Exp. No.	A-T_ext_ (°C)	B-T_pre_ (°C)	C-T_post_ (°C)	Mean Size (μm)		SD ^(c)^ (μm)
				Exp.	Pred. ^(b)^	
1	−1	0	−1	4.39	4.20	1.31
2	−1	−1	0	6.12	6.02	2.03
3	−1	1	0	2.84	2.81	1.13
4	−1	0	1	3.20	3.52	0.95
5	0	−1	−1	4.94	5.23	1.59
6	0	1	−1	2.39	2.61	0.82
7	0	−1	1	4.41	4.19	1.29
8	0	1	1	2.27	1.98	0.64
9	1	0	−1	3.09	2.77	0.77
10	1	−1	0	3.61	3.64	0.99
11	1	1	0	1.93	2.03	0.76
12	1	0	1	1.59	1.78	0.46
13	0	0	0	4.12	4.01	1.45
14	0	0	0	4.07	4.01	1.17
15	0	0	0	3.83	4.01	1.37

^(a)^ Extraction pressure was fixed at 220 bar. ^(b)^ Predicted mean size of ethenzamide crystals using Equation (1). ^(c)^ Particle size distribution represented by the standard deviation.

**Table 3:** Results of model fitting and lack of fit test in Box–Behnken design.

Model	Results	
	*R* ^2^	*p*-Value (Prob > F)
Linear	0.8619	0.0727
2FI	0.8956	0.0646
Quadratic	0.9709	0.1159

**Table 4:** ANOVA results for microparticle production of ethenzamide through RESS.

Source	Sum of Squares	Mean Square	*p*-Value (Prob > F)
Model	20.32	2.26	0.0025
A	5.01	5.01	0.0014
B	11.64	11.64	0.0002
C	1.39	1.39	0.0197
AB	0.64	0.64	0.0706
AC	0.02	0.02	0.6757
BC	0.04	0.04	0.5827
A^2^	0.62	0.62	0.0746
B^2^	0.003	0.003	0.8891
C^2^	1.04	1.04	0.0330

## Supplementary Materials

The following are available online at https://www.mdpi.com/1999-4923/12/1/42/s1, Figure S1. Effects of (a) extraction temperature (b) extraction pressure (c) pre-expansion temperature, and (d) post-expansion temperature on the mean size of RESS-processed ethenzamide. Figure S2. Particle size distribution of (a) unprocessed ethenzamide and (b) RESS-processed ethenzamide from Experiment 12. Figure S3. PXRD patterns of ethenzamide from CCDC database (CCDC number: 760137). Table S1. Operating conditions and results of RESS processing of ethenzamide.
