# Supplementary material for: Application of Box–Behnken Design to Investigate the Effect of Process Parameters on the Microparticle Production of Ethenzamide through the Rapid Expansion of the Supercritical Solutions Process
Source: Pharmaceutics. 2020 Jan 3;12(1):42. doi: 10.3390/pharmaceutics12010042 (PMC7022259; doi:10.3390/pharmaceutics12010042)
Supplement: Supplementary file 1 [file pharmaceutics-12-00042-s001.pdf]

pharmaceutics

Supplementary Materials: Application of Box–Behnken Design to Investigate the Effect of Process Parameters on the Microparticle Production of Ethenzamide through the Rapid Expansion of the Supercritical Solution Process

Yung-Tai Hsu and Chie-Shaan Su *

Table S1. Operating conditions and results of RESS processing of ethenzamide.

Exp. no.	T_{ext} (°C)	P_{ext} (bar)	T_{pre} (°C)	T_{post} (°C)	Mean size (μm)	SD (μm)
Unprocessed	----	----	----	----	15.35	4.70
S1	**45**	**220**	**130**	**10**	3.67	1.23
S2	**35**	220	130	10	3.86	1.50
S3	**55**	220	130	10	3.23	1.09
S4	45	**200**	130	10	3.32	1.25
S5	45	**240**	130	10	3.42	1.21
S6	45	220	**90**	10	6.22	2.08
S7	45	220	**110**	10	5.10	2.07
S8	45	220	**150**	10	3.33	1.29
S9	45	220	**170**	10	2.29	0.96
S10	45	220	130	**0**	3.36	1.18
S11	45	220	130	**20**	4.12	1.45
S12	45	220	130	**30**	2.77	1.09
S13	45	220	130	**40**	2.06	0.76

(a) SD: Standard deviation of the particle size distribution.

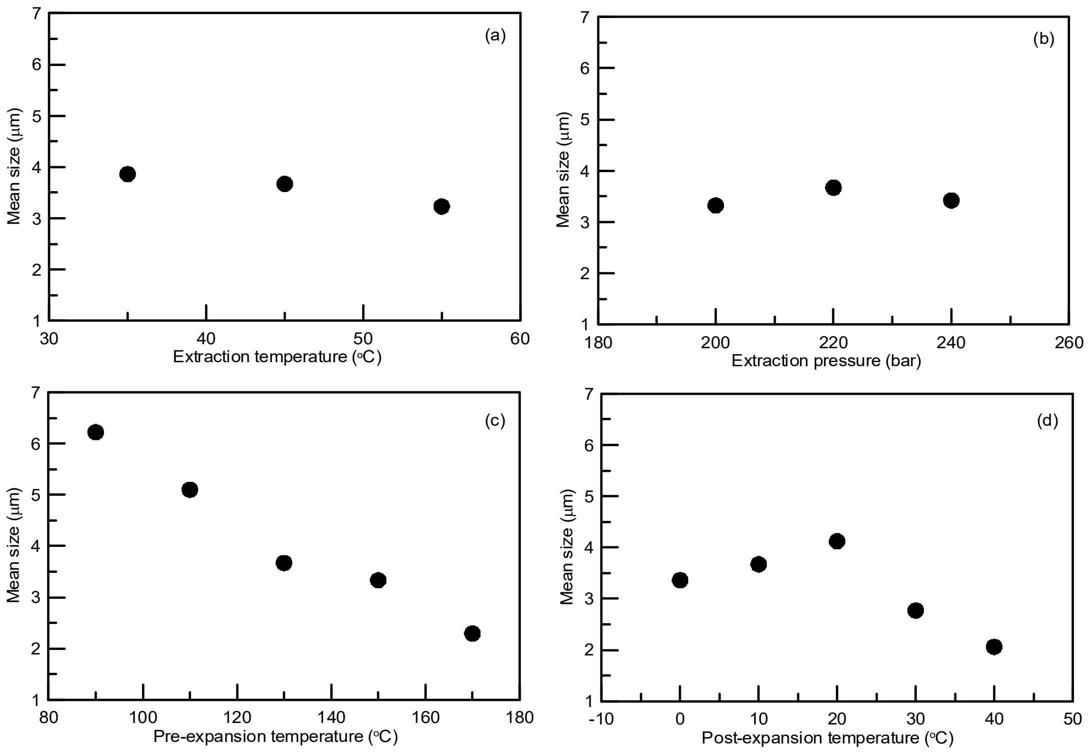

Figure S1. Effects of (a) extraction temperature (b) extraction pressure (c) pre-expansion temperature, and (d) post-expansion temperature on the mean size of RESS-processed ethenzamide.

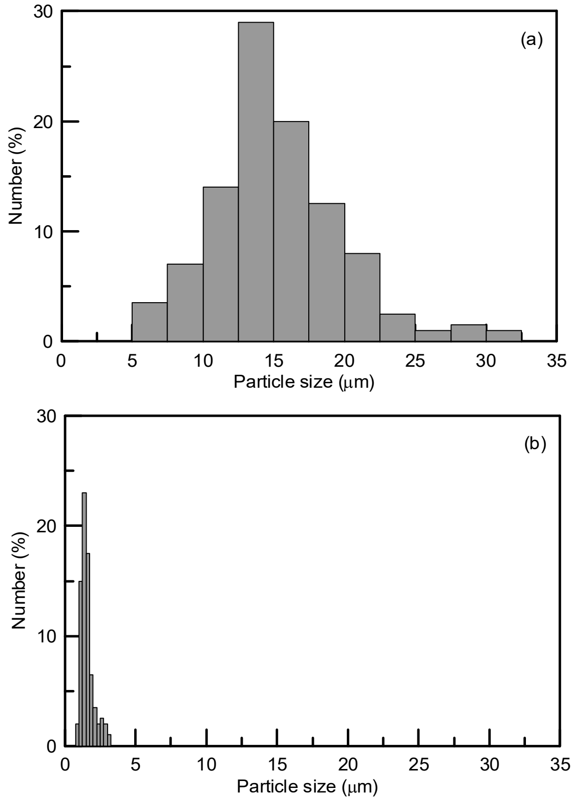

Figure S2. Particle size distribution of (**a**) unprocessed ethenzamide and (**b**) RESS-processed ethenzamide from Experiment 12.

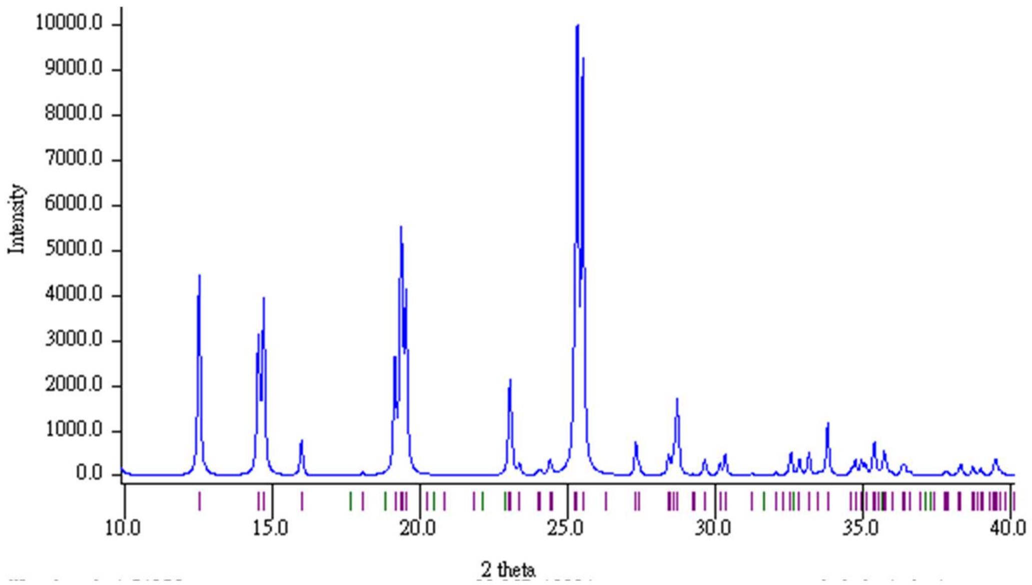

Figure S3. PXRD patterns of ethenzamide from CCDC database (CCDC number: 760137).
